# Leveraging behavioral science and artificial intelligence to support mental health in the workplace: a pilot study

**DOI:** 10.3389/fpsyt.2023.1219229

**Published:** 2023-10-19

**Authors:** Ashley B. West, Yuzhen Valerie Guo, Amy Bucher

**Affiliations:** ^1^Lirio, LLC, Knoxville, TN, United States; ^2^Behavioral Reinforcement Learning Lab, Lirio, LLC, Knoxville, TN, United States

**Keywords:** mental health, stigma, employee health, stress management, behavioral science, artificial intelligence

## Abstract

**Introduction:**

Many American employers seek to alleviate employee mental health symptoms through resources like employee assistance programs (EAPs), yet these programs are often underutilized. This pilot study explores the design of a behavioral science-based email campaign targeting engagement with stress management and mental health resources via an EAP, among employees of a large home builder in the Southeastern US.

**Methods:**

Behavioral designers created a behavioral science intervention using a multi-step design approach and evidence based behavioral strategies. For this pilot intervention, employees received either a treatment message [i.e., behavioral science message assembled and delivered via the behavioral reinforcement learning (BRL) agent] or a control message (i.e., a single generic, supportive message with a stock photo) with a call to action to utilize their EAP.

**Results:**

A total of 773 employees received emails over the course of 1 year. Engagement was high, with an 80% email open rate. Over 170 employees (22%, 159 treatment and 14 control) clicked the CTA and logged into the EAP site at least once.

**Discussion:**

This pilot study suggests that using behavioral science and artificial intelligence can improve employee usage of EAP, specifically with the intention of exploring mental health and stress management resources, compared to benchmark rates of 5% per year.

## Introduction

Supporting positive mental health and well-being is a prominent public health concern, with reducing workplace stigma around mental health being a key area of focus. Poor mental health (e.g., high stress, depression, anxiety) in the workplace contributes to increased absenteeism and presenteeism, and an overall reduction in productivity levels (1). The COVID-19 pandemic heightened the concerns for mental health across all adults in the United States (2) and increased efforts to provide support within the workplace environment as many work cultures drastically and rapidly shifted (3). However, given the complexity of mental health, interventions should consider both the individual employees as well as the workplace environment; that is, they should address both the i-frame (individual level) and the s-frame (societal level) (4).

Work is a major contributing factor to an individual’s mental well-being, and a variety of stressors such as individual level job-related factors (e.g., extended working hours, perceptions of safety or opportunities for professional growth), unfavorable psychosocial or relational workplace environments, and poor management or leadership all contribute to the likelihood of developing mental health issues as a result of work (5, 6). As many as 15% of adults worldwide have mental health conditions, with depression and anxiety specifically costing the global economy each year in both actual dollars (~1 trillion) and lost working days (~12 billion) (7). Despite the prevalence and worldwide impact of mental health on workplace productivity, employees who are struggling may refrain from speaking up or seeking supportive resources due to perceived or actual stigma (8).

The COVID-19 pandemic exacerbated employee mental health struggles by not only significantly changing the working environment but simultaneously negatively impacting daily life outside of work (9). Approximately half (51%) of employees have reported worse mental health at work since the start of the pandemic (10). These effects spared no industry or location. Global reports of negative changes in mood and stress levels (11), insomnia (12), fear, and distress (13) increased, with these negative effects being exacerbated in individuals with greater workplace seniority and among healthcare workers (14). Even several years after the start of the pandemic, the workplace has remained drastically different (e.g., prevalence of remote work) and employee mental health and well-being is still considered a high priority (3, 15).

Fortunately, most US employers offer benefits to support mental well-being (16). Among all resources and interventions, digital offerings have gained popularity in supporting employee mental health due to their ability to reduce stigma and protect anonymity at a reasonable cost (17). A common employer benefit is employee assistance programs (EAPs), designed to offer employees resources around a variety of topics, including mental health, well-being, and stress management. A 2021 survey revealed that 88% of employers reported offering EAPs and other mental health resources to their employees (16).

However, not all employee mental health resources are created equally, varying in efficacy and effectiveness as well as in level of employee engagement. Research shows it can be a challenge to find alignment between what resources employees will use and what is effective in actually improving mental health (18). For example, a systematic review of the acceptability of digital interventions to support mental health shows that individuals are largely willing to try such interventions, yet the intervention quality varies widely (i.e., not all of them are equally effective) (19). When examining EAPs specifically, research has indicated an association between EAP usage and improved work performance (20) as well as a reduction in symptoms of anxiety and depression (21). Despite these benefits, EAP utilization remains low, with data suggesting that only about 5% of employees will use their EAP benefits in a given year, with less than a quarter (~20%) using them in a 5 years period (22). It remains a challenge to find the right set of mental health offerings to provide meaningful outcomes while also engaging employees.

Prior research offers insights into some of the limitations of employee mental health interventions that may contribute to their low utilization and mixed success rates. Workers may be reluctant to participate in corporate-initiated mental health programs due to a variety of barriers including concerns about confidentiality and stigma, individual attitudes and beliefs, group dynamics and culture, and work characteristics (23–26). Mental health interventions need to be implemented in a way that makes them easy and desirable to use, including offering protection from perceived or actual stigma. Considering how to directly address stigma as well as offering private and confidential access are important considerations for employee uptake of mental health resources. The current study provided mental health support to employees via an anonymous EAP that the employee could access privately through their corporate email. Further, this study involved very inclusive eligibility (i.e., anyone with a company email address) as well as required an active opt-out (i.e., unsubscribing from messages). Interventions that require an active opt-in can create unnecessary intervention onboarding friction (e.g., diabetes behavioral intervention) (27). Similarly, opt-in strategies may negatively impact employee intervention uptake, especially for those employees most at-risk who may perceive this added friction as an additional stressor and therefore may not engage with the intervention. The current study design deployed intervention outreach without enrollment friction (i.e., no active opt-in) and without restrictions based on individual, baseline mental health. Further, the broad eligibility criteria made it such that the company norm was to receive the intervention, thereby increasing efforts to directly reduce stigma.

Stigma-related barriers can contribute to the underutilization of available mental health resources, yet employees may also face other barriers that prevent engagement with employer sponsored mental health programs. In order to increase employee engagement with mental health resources, it is critical to account for and address a variety of possible barriers. Leveraging behavioral science evidence-based tools (e.g., behavior change techniques) to address unique, individual determinants (i.e., barriers and facilitators) is an effective way to drive desired behavior change (28–30). This study leveraged behavioral science to increase participation in a workplace intervention targeting mental health and stress management. At the same time, tailored, personalized interventions are difficult to scale. Recent advancements in behavioral science and artificial intelligence have shown promise in using a behavioral reinforcement learning (BRL) agent to assemble personalized health communications and deliver them at scale across a variety of health arenas (31–33). Specifically, a BRL agent was used to deliver behavioral science-based messaging to patients overdue for their mammogram, resulting in increasing the number of scheduled and attended screenings in a population overdue for this prevention visit (31). Artificial intelligence has also been used to deliver personalized messages prompting individuals to complete their COVID vaccine series (32). Reinforcement learning specifically offers the ability to increase the frequency of a desired behavior based on data feedback (34). This study combined a BRL agent with behavioral science to predict individual determinants and prompt employees to engage with their corporate EAP and explore its mental health resources.

The success and sustainability of employee mental health interventions depend heavily on their integration into the working environment, situational factors, and active, sustainable involvement from employees. Research specifically finds that increased opportunities for worker participation in programs and more control over how to do so is associated with improved outcomes (35). Similarly, a meta-synthesis of research on workplace mental health interventions found scheduling flexibility and accommodating resource utilization during work time were positively associated with successful implementations (23). Finally, the COVID-19 pandemic severely restricted the ability for employers to provide in-person mental health support, heightening the need for flexibility in resource access and utilization. The current intervention offered unlimited, 24 h remote access to mental health resources via an online EAP portal shortly after the COVID-19 pandemic began.

This pilot study explored the design of a behavioral science-based intervention to drive increased engagement with mental health resources, including stress management tools, via an EAP among employees of a large home building company in the Southeastern US. The current intervention was informed by the gaps from past literature and aimed to address these systemic and contextual challenges. Contextual factors such as the workplace environment, employee social interactions, availability of breaks and strategic recovery, and other organizational considerations were addressed through audit-driven behavioral science recommendations that complemented the email intervention.

## Methods

### Participants

Intervention participants were 773 corporate employees from a large home building company in the Southeastern United States. Employees were eligible for the intervention if they had a company email address. There were no exclusion criteria for this study, and, because this intervention was implemented as part of a mental health initiative at the described company, no demographic data were collected.

### Procedures

#### Behavioral science-based intervention design

In order to support employee mental health, an email campaign was designed to encourage use of mental health resources via a workplace sponsored EAP, delivered through an online portal.

Behavioral designers were doctorally trained in the fields of public health, behavioral science, and digital health intervention development and deployment. Designers had 5+ years of experience designing behavioral science infused interventions for a wide variety of health topics (e.g., preventive cancer screenings, physical activity, sedentary behavior, hydration).

Behavioral designers followed a multi-step intervention development approach that included incorporating behavioral strategies and design practices from behavioral economic principles, the COM-B Model, the Behavior Change Wheel, and the Theory and Technique Tool (28, 29, 36). These behavioral frameworks and tools were specifically chosen because they not only explain how health behavior change occurs but also describe the mechanisms through which behavior change is possible. The COM-B Model allows interventionists to account for multiple levels of influence (i.e., capability, opportunity, motivation) that can impact whether an individual takes the desired health action. The Behavior Change Wheel and Theory and Technique Tool both describe strategies for maximizing those levels of influence and driving the individual to positive behavior change. Specifically, the Theory and Technique Tool provides interventionists with an online tool for selecting behavior change techniques from a list of 93 techniques (e.g., information about health consequences, social support, incentives). This tool allows interventionists to explore each behavior change technique’s level of evidence for successfully driving behavior change across 26 mechanisms of action for behavior change. Despite the reliability and reproducibility associated with these models and tools (36), they do have limitations. For one, often multiple behavioral techniques from the Theory and Technique Tool are shown to be successful in driving or prompting the desired health behavior, and therefore the designer must lean on their expertise and understanding of the target population and other contextual factors to prioritize their use. Further, specific effect sizes of each of these behavioral techniques can be difficult to calculate and thus understanding how much behavior change can be expected to be attributed to any single technique is complicated. Often times, intervention designs are better positioned to highlight success at a more global level (i.e., did the intervention as a whole result in the desired behavior change?).

The intervention was designed in four major steps. First, behavioral designers compiled a list of behavioral economic based strategies that have been shown to drive engagement with health communications (e.g., social proof, mere measurement effect). Then, behavioral designers conducted a literature review to identify the behavioral determinants associated with engaging with mental health resources via a workplace sponsored portal, such as an EAP. A list of these determinants can be found in Table 1. Each determinant (*n* = 10) was then paired with a mechanism of action (MoA) (e.g., skill, intention, social influences). These MoAs describe how behavior change techniques (e.g., instruction on how to perform behavior, information about health consequences, social comparison) are used to drive behavior change. Finally, designers used the Theory and Technique Tool to select behavior change techniques shown to have a positive effect on behavior change through the identified mechanisms of action, either by helping an employee overcome a barrier to engaging with mental health resources or facilitating employee usage of the EAP resources. As an example, if the behavioral determinant is mental health literacy, where an individual’s ability to recognize poor mental health can predict whether they will seek out and engage with mental health resources, the mechanism of action through which behavior change can occur could be skill. In other words, improving an individual’s skills with identifying mental health needs and seeking out resources increases their ability to complete the target behavior (i.e., log into EAP portal and explore mental health resources). The behavior change technique instruction on how to perform behavior can be used in intervention content to build these skills. For example, the intervention content that leveraged this behavior change technique in this study described how to log into EAP and what resources the person would have access to.

**Table 1:** List of behavioral determinants for engaging with mental health resources (including via a workplace sponsored portal such as an EAP).

Individual	Social	Environmental
Desire for anonymity (e.g., EAPs that are perceived as safe, confidential, and secure facilitate more employee engagement)	Social support from an individual’s network (e.g., family, friends, co-workers, health providers) can impact engagement	Convenience level (e.g., ease of access in general as well as flexibility in when and where the EAP can be accessed can facilitate employees seeking out and using mental health resources)
Mental health literacy (e.g., an individual’s ability to recognize poor mental health can predict whether they will seek out and engage with mental health resources)	Perceived or actual stigma from one’s social network regarding mental health can inhibit engagement	Lack of time to engage with mental health resources
Adherence to digital tools and programs that support mental health and well-being facilitates continued engagement with these types of resources		
Attitudes (positive or negative) towards mental health support, including resources like EAPs		
The desire to independently self-manage mental health can inhibit engagement with mental health resources		
Lack of awareness and knowledge about the fact that mental health resources exist and where and how to access them blocks engagement		

The four-step design process outlined above yielded a suite of unique behavioral strategies (*n* = 20) that either drive initial engagement with the intervention email messages or prompt the employee to complete the desired behavior (i.e., log into their EAP portal and explore mental health resources). A comprehensive list of the behavioral strategies leveraged in this intervention can be found in Table 2.

**Table 2:** Behavior change strategies to either drive engagement or completion of the desired behavior.

Initial engagement strategies	Prompts for action (i.e., behavior change techniques)
Curiosity	Anticipated regret
Gain frame	Behavioral practice/rehearsal
Implementation intentions	Credible source
Loss aversion	Comparative imagining of future outcomes
Power of free	Goal setting
Prosocial	Information about health consequences
Reduce friction	Instruction on how to perform behavior
Self-efficacy	Salience of consequences
Social proof	Social comparison
Trusted messenger	Social support (emotional)

The behavioral designers then worked with content creators to build a library of behavioral science infused email messages. Each message was comprised of a subject line, preheader, headline, body copy, a hero image, and a call-to-action (CTA) button. Further, each behavioral strategy was operationalized three different ways, leading to an intervention message library with 30 content items targeting engagement (i.e., subject lines and preheaders) and 30 content items directly prompting the desired action (i.e., headlines, body copy, hero images).

The intervention CTA buttons in each email (*n* = 2) would automatically log recipients into their employer’s EAP if clicked. From there, the employee could explore a variety of resources to support positive mental health including stress management tools.

#### Intervention delivery

The intervention was delivered via an artificial intelligence, BRL agent algorithm. The BRL agent utilized for this intervention is based on best practices from the behavioral reinforcement and machine learning literature (37–39). First, behavioral designers established the target behaviors of logging into EAP and using specific resources, with secondary target behaviors of opening and interacting with messages. The BRL agent (algorithm) was programmed with a reward function that prioritized the primary target behavior of logging into EAP with smaller rewards when recipients completed the secondary target behaviors of opening and interacting with the messages themselves. The agent facilitates the delivery of tailored, personalized content to each recipient by learning what messages elicit desired behaviors through feedback (e.g., engagement data such as opening the communication), as well as demographic and contextual factors when available. For this intervention, prior information about the recipient (i.e., employee) was limited and therefore the learning agent relied only on behavioral feedback to select intervention content. Specifically, the BRL agent algorithm was trained to understand what combination of subject lines and body copy (with hero images) maximized the likelihood that the employee would engage in the specified target behaviors: open the email and engage with content, and more importantly, click on the CTA and log into the EAP to explore mental health resources. The data indicating whether these behaviors had occurred was passed back to the BRL, allowing it to learn over time about intervention engagement and subsequently improve and adapt email message compilation for each employee. For example, the BRL agent may learn that email content detailing the benefits of EAP for mental health and stress management resources is associated with higher intervention engagement, and therefore, may prioritize sending content that focuses on these incentives in future messaging.

For this intervention, the BRL agent assembled each of the content items, described above in the intervention design section, into 1,800 possible email combinations. The agent then considered several data inputs (e.g., prior engagement with messages and participation in the desired behavior) to determine which email combination should be delivered to each employee, allowing for the delivery of personalized emails based on individual employee characteristics.

Employees received the intervention via their company email address. Intervention material was delivered following a strategy that allowed for regular intervention exposure while also guarding against notification fatigue. Specifically, employees could receive one intervention email message each week for the first 3 weeks, and then a message every other week thereafter for a total of 12 weeks. The maximum number of intervention messages an employee could receive was seven. Employees could opt out of the intervention communications by clicking the unsubscribe button at the bottom of the email. This intervention was delivered from February 2021–February 2022.

#### Treatment and control conditions

This pilot study was designed to establish the feasibility of delivering a behavioral science-based intervention to employees as part of the company’s expanding mental health initiatives in the wake of the COVID-19 pandemic. Therefore, the study was not powered to detect statistical significance between treatment and control groups. Further, given that this study was a pilot study conducted as part of the described company’s efforts to increase accessibility to mental health resources, priority was focused around providing as many employees as possible with the treatment content. In the interest of maximizing the effectiveness of the overall intervention, a larger percentage of the population received the treatment messages (90% vs. 10%). Employees were randomly selected to receive either the treatment messages or control message, and all employees were equally eligible to be placed in either condition at the start of the study.

**Figure 1:**
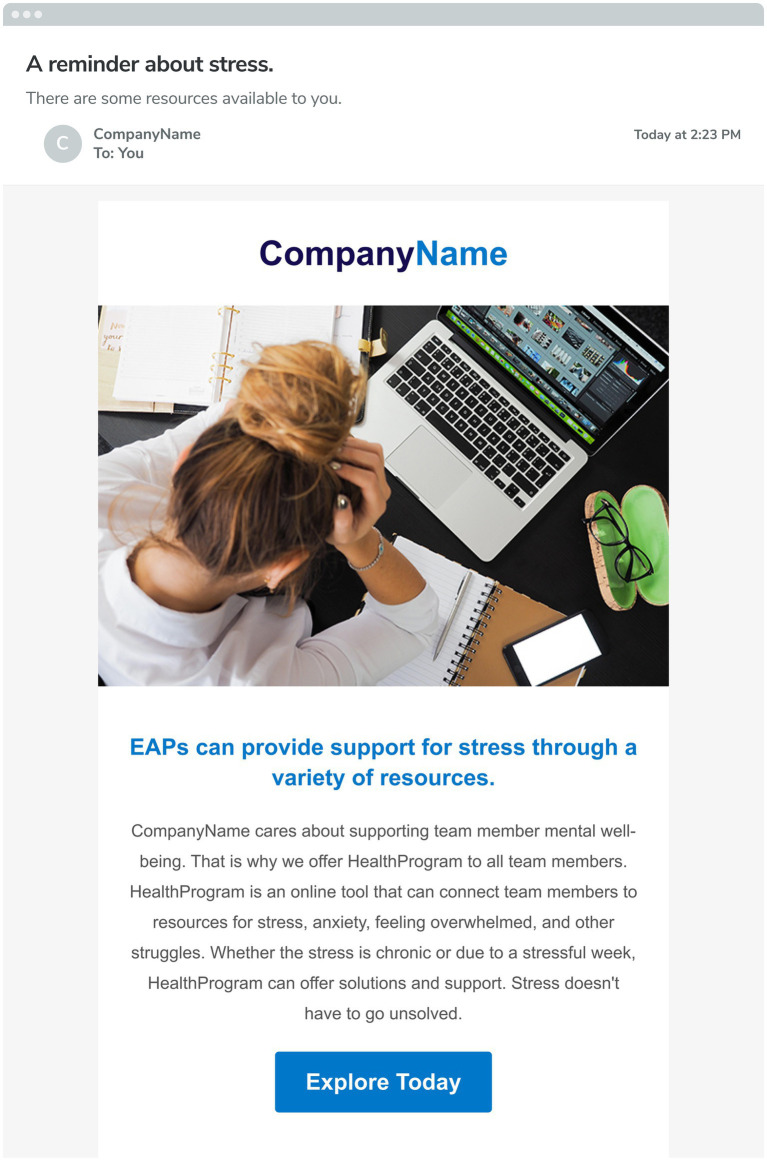
Example of an intervention message compiled by the BRL agent.

**Figure 2:**
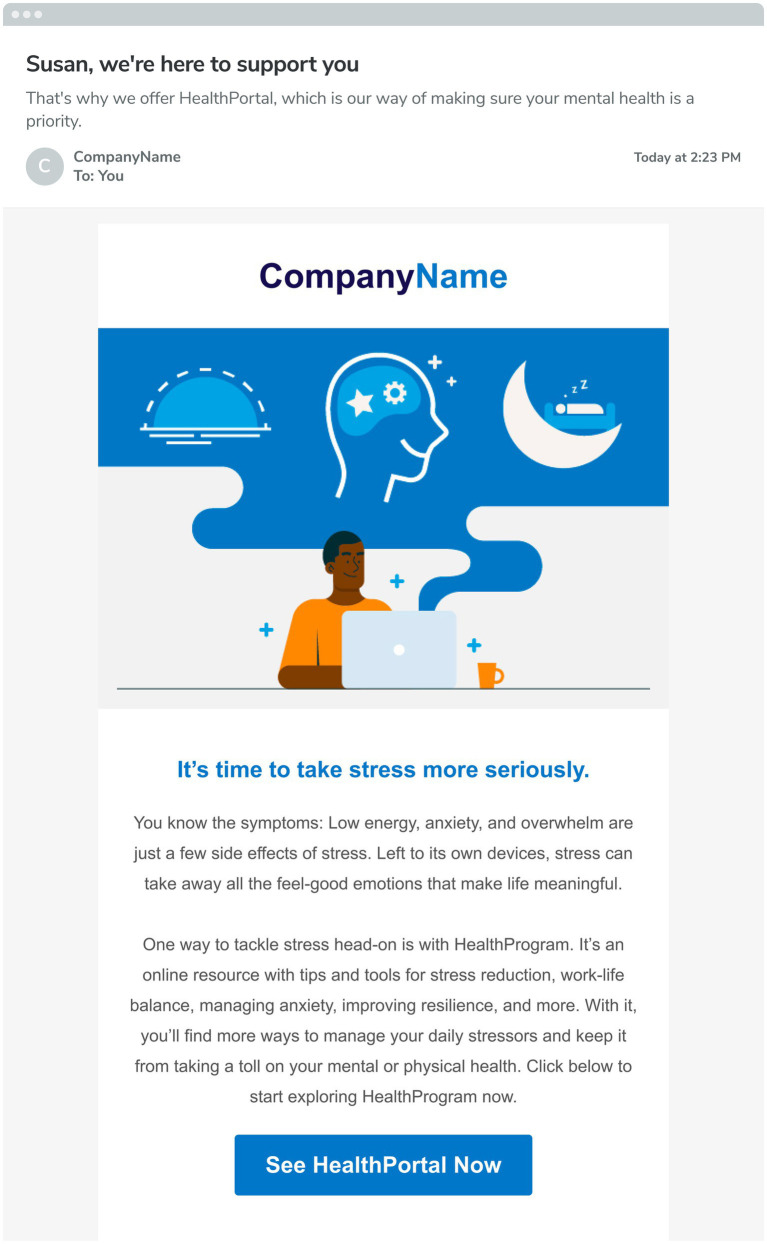
Single control message that includes generic, supportive content with a stock photo.

All messages included the components of a subject line, preheader, headline, body copy, hero image, and CTA button. Employees were randomly selected to receive a treatment message (i.e., behavioral science message assembled and delivered via the BRL agent) or control message (i.e., a single generic, supportive message with a stock photo). The control message was written to express what the employee would gain from utilizing the EAP for mental health support, a common tactic used in the company’s existing marketing materials. An example of an intervention email and the control email can be found in Figures 1, 2, respectively.

### Measures

Employee engagement with the intervention was defined as an employee opening the email that was promoting EAP utilization. The email prompt was considered successful if the employee clicked the CTA in the email and logged into the EAP website. The BRL agent tracked these opens and clicks in order to inform future email combinations that should be delivered to each employee.

### Transparency and openness

This intervention was implemented as part of a mental health initiative at the described company to increase employee mental health support in the wake of the pandemic. Therefore, this study and the analysis plan were not pre-registered and de-identified data are not publicly available.

## Results

Participants (*N* = 773) were corporate employees, both men and women, at a large home building company in the Southeastern US. Participants held various traditional, corporate roles including but not limited to call center support, home insurance, and employee benefits/human resources. All participants were eligible to receive the email campaign from February 2021 through February 2022. Engagement with this pilot intervention was high, with the majority of participants (79.9%) opening the email messages (80.3% for treatment and 76.8% for control). Throughout the study period, only four people (0.05%) opted-out of the intervention (i.e., unsubscribed from the email messages). Across the intervention year, over 170 employees (22%) completed the desired behavior at least once (i.e., clicked the email CTA and logged into the EAP site to explore mental health resources). Of these 170 employees, the majority received the treatment content (i.e., 159 employees vs. 14). Finally, any behavioral insights and strategies identified during peer-reviewed and product research that were not suitable for the described pilot intervention yet were still relevant to supporting employee mental health (e.g., recommendations for improving workplace environment and co-worker interactions), were summarized in a report and delivered to the organization with implementation recommendations.

## Discussion

This study used a BRL agent to deliver a behavioral science-based intervention for employees at a home building company, targeting engagement with mental health resources through an EAP online portal. The intervention had broad eligibility criteria that did not rely on baseline mental health status, deployed an active opt-out to reduce intervention onboarding friction, and was piloted during the height of the COVID-19 pandemic.

Research into the barriers experienced by employees also led to a comprehensive report containing recommendations for how the organization could support employee mental health alongside the behavioral email campaign. Implementing organizational-or team-level strategies may help improve the impact of individual-level interventions such as a behavioral science-based intervention used in this study by creating circumstances favorable to EAP use. The specific recommendations were intended to provide organizational support around mental health initiatives, like the email campaign, and also require a minimal implementation budget.

While this pilot intervention was not powered to show statistical significance, the preliminary results are promising. The EAP engagement rates associated with the intervention compare favorably with national data showing approximately 5% of employees access EAP in a given year (22). During the intervention period, 22% of the participating employees accessed EAP at least once. The emails themselves also received strong engagement rates, with 80% of the messages being opened and a very low unsubscribe rate of 0.05%.

Using a BRL agent to personalize the selection and unique combination of behavioral strategies likely drove improvements over more generic email campaigns. There is a general issue of low employee awareness of available resources such as EAP and the benefits they provide (40). A corporate email blast with information about EAP could easily be ignored or skimmed by recipients. Personalized communications, on the other hand, have been shown to receive greater attention and improve information retention (41). Additionally, by focusing on one specific barrier and behavioral strategy in the body of the email, the real estate within the message could be used not to just to inform the recipient of the existence of the EAP resource, but also address specific hesitations they may have about using it.

Personalization enabled the intervention to address the breadth of barriers employees may experience to using EAP resources without presenting employees with content that is not relevant to their particular concerns. The behavioral science-based intervention addressed 10 high level barriers to EAP usage through three different messages per barrier. Each employee only saw messages likely to be meaningful to them based on their past interaction patterns. This presentation style reduces user burden by delivering pointed, relevant content, and may support health equity by facilitating the inclusion of barriers experienced by under-served groups (32).

Emailing also may have been an effective channel for this outreach because it offers privacy and the ability for recipients to read and react to messages at a time of their choosing. Given that stigma is a reason why many employees refrain from using workplace mental health resources, it is important to communicate in a way that can be kept private and confidential from others.

Offering a digital suite of EAP resources (e.g., stress management tools) may have also promoted engagement. While digital support tools may not be sufficient to address all mental health concerns, many have been associated with significant reductions in symptoms and distress (42), and increases in productivity (43). The current intervention offered a logical way for employees to take immediate action by clicking to log into the EAP portal and explore its offerings. Compared to attending a health fair, phoning a hotline, or setting an appointment with a counselor, this is a low-friction behavioral loop. Future research should examine how to introduce employees with more significant mental health needs to higher touch services as part of the EAP experience.

### Limitations and future research

Results from this study may not generalize to non-corporate employees. This intervention was delivered to employees with a company email address, limiting outreach only to employees working in the corporate setting. However, employees who work in blue collar jobs such as within the manufacturing and home building roles did not have a company email address. Future research should explore opportunities for increasing access to mental health resources for employees who work outside of corporate offices. Further, the stress and other mental health issues faced by these employees may differ from those experienced by those in an office setting. Future research should explore what determinants and behavioral strategies may be more applicable to different employee roles.

Demographic data were not collected as part of this pilot study. Therefore, conclusions about the diversity of the participant sample as well as any impact of various demographic factors on engagement with the intervention is unknown. Future research should deploy this intervention design among a diverse sample of corporate and non-corporate employees.

This pilot implementation was designed to establish feasibility and meet an immediate organizational need to support employees, and so was not sufficiently powered to detect significant differences between the control and treatment groups. In addition to a larger and appropriately powered replication of the current work, future research should determine which behavioral strategies can improve employee engagement with mental health resources when compared to traditional tactics such as education about available benefits. Future research can also explore how to drive not just engagement but also utilization of EAP benefits.

This intervention highlighted the challenges in tracking both how employees are engaging with mental health resources and whether there is mental health improvement in the workplace. For instance, it is possible more than 22% of employees logged into their EAP as a result of the intervention (i.e., an employee could have logged into EAP without using the CTA button and therefore would not be reflected in the current count). Further, it is unclear if employees logged into the EAP site once or multiple times to engage with the mental health resources over the course of the yearlong intervention period. Finally, this study was not able to capture how employees utilized the resources once they logged into the EAP site (e.g., how many resources were explored, was anything downloaded or printed out). Outcomes associated with use of the EAP resources, such as any reductions in self-reported stress levels or adoption of new coping strategies, were also not measured. Future research should explore how best to measure and capture improvement in employee mental health and sustained usage of these digital resources, particularly when engagement with resources within an EAP may need to consider anonymity and privacy concerns.

Finally, there is an opportunity to build on the pilot intervention for a more robust implementation. This would include not just extending intervention access across the company, but also reviewing the performance of specific behavioral strategies in the pilot implementation in order to refine the message content. It would also be worth revisiting the barriers addressed in the intervention, given the speed with which workplace cultures have changed post-COVID-19. Future research should explore how addressing mental health stigma in the workplace from a variety of angles (i.e., individual vs. interpersonal vs. environmental influences) and message content could differentially impact employee mental health and well-being.

## Conclusion

Employee mental health is an issue of significant importance to employers, given its pernicious effects on employee attendance, productivity, and well-being. Employers have invested in tools, such as EAPs, that can help address employee challenges of stress, anxiety, depressive symptoms, etc. But even when employees are aware they have access to these programs, barriers including stigma may prevent them from engaging. Interventions that personalize outreach to specifically address the unique barriers each employee has to using mental health interventions can increase engagement. Further, it is critical to not only consider the individual level, but also the workplace context in which these resources are delivered. The best results will come when employers reshape the work environment to reduce stigma while simultaneously connecting employees to the tools that will help them with mental well-being and resilience.

## Data availability statement

The original contributions presented in the study are included in the article/supplementary materials, further inquiries can be directed to the corresponding author.

## Ethics statement

The studies involving humans were approved by Solutions IRB, identifier Subject: Protocol #2021/05/28. The studies were conducted in accordance with the local legislation and institutional requirements. Written informed consent for participation was not required from the participants or the participants’ legal guardians/next of kin in accordance with the national legislation and the institutional requirements.

## Author contributions

ABW: contributed to the design of the behavioral nudging campaign described in the manuscript, supported the acquisition of the data, responsible for conceptualizing the topic for the manuscript and interpretation of the data, and drafted the manuscript and critically revised it. As first author and corresponding author, ABW agrees to be accountable for all aspects of the work in ensuring that questions related to the accuracy or integrity of any part of the work are appropriately investigated and resolved. YG and AB: drafted portions of the manuscript and critically revised it and provided approval for publication of the content. AB: supported conceptualizing the topic for the manuscript and interpretation of the data as well as supported data analysis. All authors contributed to the article and approved the submitted version.
